# Lupeol‐Loaded Liposomes: Triple‐Negative Breast Cancer (TNBC) Cytotoxicity and In Vivo Toxicological Evaluation

**DOI:** 10.1002/cbdv.202500249

**Published:** 2025-07-10

**Authors:** Daniel Crístian Ferreira Soares, Daniel Bragança Viana, André Luis Branco de Barros, Sued Eustáquio Mendes Miranda, Daniela Sachs, Eduardo Henrique Martins Nunes, Eryvaldo Sócrates Tabosa do Egito

**Affiliations:** ^1^ Laboratório de Bioengenharia Universidade Federal de Itajubá Itabira Minas Gerais Brazil; ^2^ Faculdade de Farmácia, Departamento de Análises Clínicas e Toxicologias Universidade Federal de Minas Gerais Belo Horizonte Minas Gerais Brazil; ^3^ Universidade Federal de Itajubá, Instituto de Física e Química Itajubá Minas Gerais Brazil; ^4^ Escola de Engenharia, Departamento de Engenharia Metalúrgica e de Materiais Universidade Federal de Minas Gerais Belo Horizonte Minas Gerais Brazil; ^5^ Departamento de Farmácia Laboratório de Sistemas Dispersos‐LaSiD Universidade Federal do Rio Grande do Norte Natal Rio Grande do Norte Brazil

**Keywords:** lupeol toxicology evaluation, lupeol‐liposomes, selective cytotoxicity, triple‐negative breast cancer treatment

## Abstract

Lupeol is a natural product commonly found in many vegetables and fruits in significant quantities. Previous studies have demonstrated the relevant activity of lupeol against various tumor cell lines, suggesting that the natural product could be a promising agent for cancer treatment. Due to bioavailability requirements, lupeol presents a challenge for internal administration in therapeutic doses. Thus, the development of pharmaceutical formulations, particularly nanostructured systems, could represent an important alternative to improving the bioavailability of the molecule. This study developed and characterized a liposomal formulation loaded with lupeol, exhibiting substantial cytotoxic, cytostatic, and apoptotic profiles against the MDA‐MB‐231 tumor cells, a triple‐negative human breast cancer subtype. Toxicological in vivo evaluations revealed a nonrelevant toxic profile against healthy mice. Considering all the results obtained, the present study revealed important potentialities of the liposomal system, constituting a potential therapeutic alternative against triple‐negative breast cancer.

## Graphical abstract

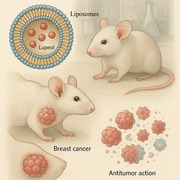

## 1 Introduction

Drug discovery processes date back to the ancient ages when different sources of inorganic and organic compounds were extensively used to treat a wide variety of diseases. In modern times, following a scientific approach that started around the late 1800s, natural products have played a relevant role as inspiring sources for synthesizing bioactive compounds such as polysaccharides, alkaloids, and peptides [1, 2, 3]. Different classes of natural products have been used as a source of antitumor agents, among which the pentacyclic triterpenes can be highlighted [4, 5, 6, 7]. These terpenes are natural products that modify critical intracellular pathways such as the nuclear factor kappa B, toll‐like receptors, PI3K/Akt/mTOR activators, and transcription factor 3 of tumor cells [8, 9, 10]. In addition, different pentacyclic triterpenes can trigger apoptotic processes in various tumor cell lines [11, 12, 13]. However, the influence of these molecules on other signaling pathways is still unknown, and further studies shall be conducted to expand the knowledge on the potential use of these compounds as therapeutic agents.

Some triterpenes have been approved for clinical trials, and recent studies have demonstrated effective results of these molecules in treating various diseases, including cancer. In particular, the lupane‐type triterpenes have shown superior activity against several cancer cell lines. For example, Pyo et al. [14] evaluated Betulin's efficacy against lung cancer. The results showed that the triterpene could induce apoptosis in the human lung cell line A549, reaching an IC_50_ of 20 µM, comparable to cisplatin (25 µM). A few years later, Li [15] studied the role of the same triterpene in the AMPK (AMP‐activated protein kinase) activation with a relevant reduction in the mTOR and S6 kinase phosphorylation using the same lung cancer cell line.

**FIGURE 1:**
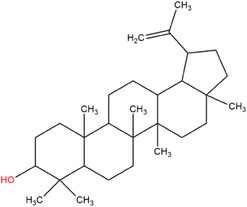
Lupeol's molecular structure. *Source*: Image extracted from PubChem, available on https://pubchem.ncbi.nlm.nih.gov.

Another important example of a lupane antitumor triterpene is lupeol. Recently, Zhang [16] demonstrated that lupeol could inhibit the actions of triple‐negative breast cancer (TNBC) cells by inducing the autophagy process. These results and many previous studies have shown similar antitumor activity against various tumor cell lines, suggesting that lupeol could be a promising cancer treatment agent. Lupeol is a pentacyclic triterpene that can be found in significant amounts in vegetables such as tomato, white cabbage, cucumber, and pepper and in fruits such as mango, strawberry, and red grape, as well as in different medicinal plants available in various countries [17, 18, 19]. Their molecular structure contains only one hydroxyl radical, located on ring A, which contributes to its low solubility in water, resulting in a Log *p* value of 7.67 (Figure 1) [20, 21]. This property represents a fundamental challenge for the internal administration of this molecule, and the development of pharmaceutical formulations could be an important alternative to improve the bioavailability of lupeol.

Some nanostructured formulations based on polymeric nanosystems have ultimately been proposed to carry lupeol. However, unlike polymeric nanosystems, liposomal vesicles are well‐established as one of the lowest immunogenicity carrier systems, mainly due to their natural lipid composition and incomparable versatility in encapsulating hydrophobic agents. To the present day, the encapsulation of lupeol in liposomal formulations can be found in only a few studies, and specifically, applications regarding breast cancer remain almost unexplored. For example, a search performed on the Scopus platform (accessible at https://www.scopus.com/search/form.uri?display=basic#basic), focusing on the last 10 years, using the keywords “lupeol and liposomes” yielded a total of 14 published papers. The most related works were observed in 2020 and 2025, with only four publications (data from December 2024). Although the antitumor activity of lupane‐type triterpenes has been extensively investigated in recent years, the use of liposomal formulations as carriers for lupeol remains almost unexplored. Thus, this work focused on developing, purifying, and characterizing a novel liposomal formulation encapsulating lupeol, aiming to expand the repertoire of available anticancer agents, particularly those related to the TNBC treatment.

## 2 Results and Discussion

Liposomes were prepared using two different methods: (i) lipid hydration method, initially described by Bangham et al., and (ii) reverse phase evaporation (REV) method [22, 23]. Liposomes were prepared in two variants: blank liposomes (TSL‐BLK) and liposomes loaded with lupeol (TSL‐LUP), which were prepared according to previously described procedures conducted by Lopes et al. [24], with some modifications. The formulations were obtained as a milky dispersion, similar to the blank liposomes (control). Furthermore, the formulations exhibited a uniform phase, indicating a homogeneous aspect. In the next step, the prepared formulations were characterized by dynamic light scattering (DLS) and zeta potential techniques. The data obtained for REV and Bangham methods are shown in Table 1. TSL‐LUP05 and TSL‐LUP01 formulations (lupeol at 500 µg mL^−1^ and lupeol at 1000 µg mL^−1^, respectively), prepared through REV methodology, showed an average hydrodynamic size of around 119 nm and polydispersity index (PDI) of around 0.2. However, the formulations obtained through Bangham methodology displayed PDI of approximately 0.3 and an average size of around 125 nm (median).

**TABLE 1:** Average size, polydispersity index, and zeta potential results of TSL‐LUP01, TSL‐LUP05, and blank liposome samples for the reverse phase evaporation and Bangham methodologies.

Methodology	Sample	Average size (nm)	PDI	Zeta potential (mV)
**REV**	Blank	105 ± 7	0.21 ± 0.03	−5.2 ± 0.4
	TSL‐LUP05	125 ± 8	0.22 ± 0.01	−5.7 ± 0.7
	TSL‐LUP01	127 ± 5	0.26 ± 0.02	−5.8 ± 0.6
**Bangham**	Blank	112 ± 6	0.31 ± 0.03	−5.0 ± 0.4
	TSL‐LUP05	135 ± 5	0.30 ± 0.01	−5.4 ± 0.6
	TSL‐LUP01	142 ± 3	0.31 ± 0.02	−5.3 ± 0.6

Abbreviation: PDI, polydispersity index.

The set of results indicates that lupeol, added to the hydrophobic part of the phospholipid bilayer, changes the characteristics of the vesicle in terms of average size, achieving around 16%–18% increase in size. Liposomes are mainly carriers for hydrophilic molecules encapsulated within the vesicle's core. The addition of hydrophobic molecules across the phospholipid bilayers could induce a relevant enlargement of the vesicular structure, leading to a perceptible increase in terms of size. Caldeira de Araújo Lopes et al. also observed a similar behavior when preparing a liposomal formulation based on ursolic acid, a highly hydrophobic pentacyclic triterpene. The results showed an increase in size of up to 15% compared to the blank formulation [25, 26].

Regarding the zeta potential, the chemical composition of the prepared liposomes reflected an almost neutral zeta potential. As expected, this value may raise concerns about the system's stability. To better understand these characteristics, a storage stability study was performed in this work, and the results obtained can help us understand the system's behavior over time.

### 2.1 Storage Stability Evaluation

**FIGURE 2:**
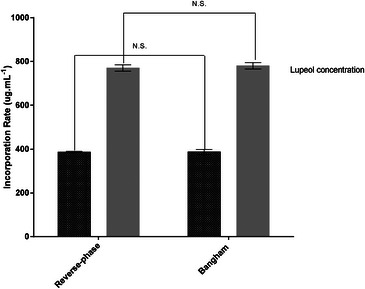
Incoporation rate of lupeol in liposomes prepared by different approaches (reverse‐phase evaporation and Bangham methods) at 0.5 mg and 1.0 mg. mL^−1^.

The formulation stability was evaluated in the samples stored at 10°C for 84 days, and the obtained data are available in Figure 2. Aliquots of these formulations were taken and analyzed, and the results obtained at different times revealed that both prepared formulations had a suitable average hydrodynamic diameter and PDI, which remained stable even after 56 days, where no significant statistical differences were observed (*p* < 0.01). However, after 70 days, changes were observed in the TSL‐LUP01 formulation, evidenced by a noticeable increase in the effective diameter of the vesicles. In contrast, TSL‐LUP05 samples remained unchanged throughout the entire study period. Similar behavior was observed when the PDI parameter was evaluated, and the values remained almost the same for 56 days. After 70 days, the results revealed a significant change in both formulations in terms of PDI, with a relevant increase in value that can be associated with the increase in size with a probable phenomenon related to self‐aggregation, coalescence, flocculation, and precipitation [27, 28]. Although the formulations developed have demonstrated relevant in vitro stability for 70 days, the drug release behavior and kinetic studies in different biological media could reveal additional data and contribute to expanding the physicochemical knowledge of the developed systems.

### 2.2 Encapsulation Efficiency (EE) Study

**FIGURE 3:**
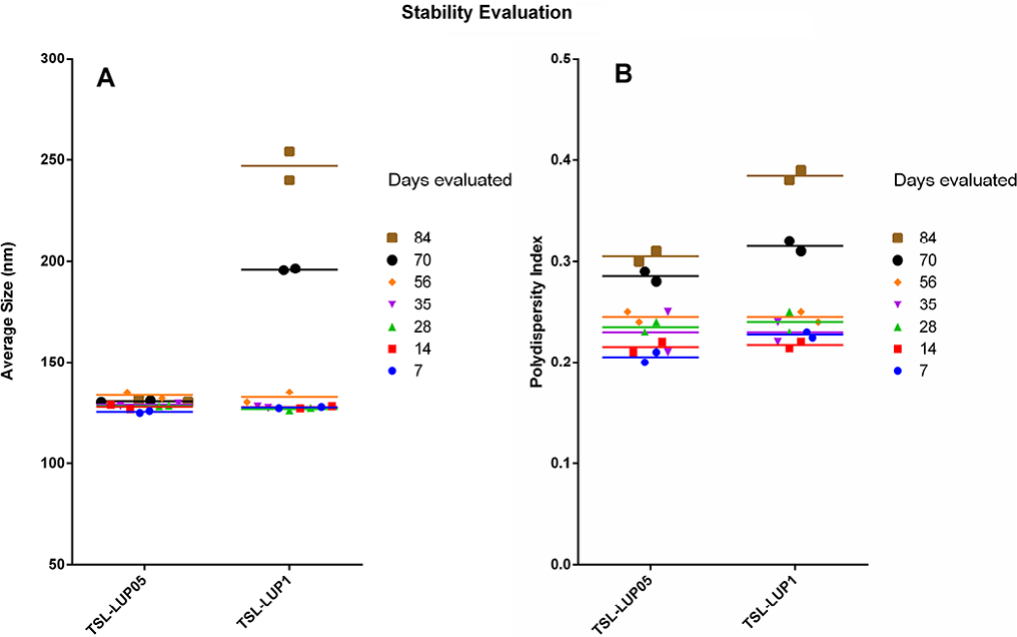
Storage stability evaluation of prepared formulations (TSL‐LUP05 = lupeol at 0.5 mg. mL^−1^ and TSL‐LUP‐01 = lupeol at 1.0 mg.mL^−1^ at different times ranging from 7 to 84 days). (A) Liposome s temporal behavior in terms of average size. (B) Liposome s temporal behavior in terms of polydispersity index (PDI). The samples were stored at 10 C, and the analyses were conducted through the DLS technique.

Lupeol's EE was determined by high‐performance liquid chromatography (HPLC) analysis for the two liposome preparation methods used (REV and Bangham methodologies). Furthermore, the EE was evaluated considering the two different concentrations of lupeol (0.5 and 1 mg mL^−1^ to 2.34 mM and 1.17 mM). The data obtained are available in Figure 3. When the liposomes were prepared using a lupeol concentration of 0.05% w/v, a significant amount of lupeol was entrapped, reaching 386.10 ± 5.6 µg mL^−1^. However, when using a concentration of 0.1% w/v, a substantial improvement was obtained, with the total amount of encapsulated lupeol reaching 775.2 ± 9.4 µg mL^−1^.

These values correspond to an EE (%) of about 77% for lupeol concentrations tested. Considering both employed methodologies, no significant differences were observed regarding lupeol encapsulation. However, the authors chose the TSL‐LUP01 formulation to continue the morphological characterization and in vitro and in vivo studies to avoid using organic solvent (ether). Regarding the lupeol EE, the data obtained in this study are in accordance with previous studies available in the literature. For example, similar data were found by Zhang and Zhang et al. who evaluated the in vitro and in vivo antitumor effects of lupeol‐loaded in galactosylated liposomes and lupeol PEGylated liposomes, which reached EE (%) of about 83% and 86%, respectively [29, 30].

### 2.3 Morphological Characterization

**FIGURE 4:**
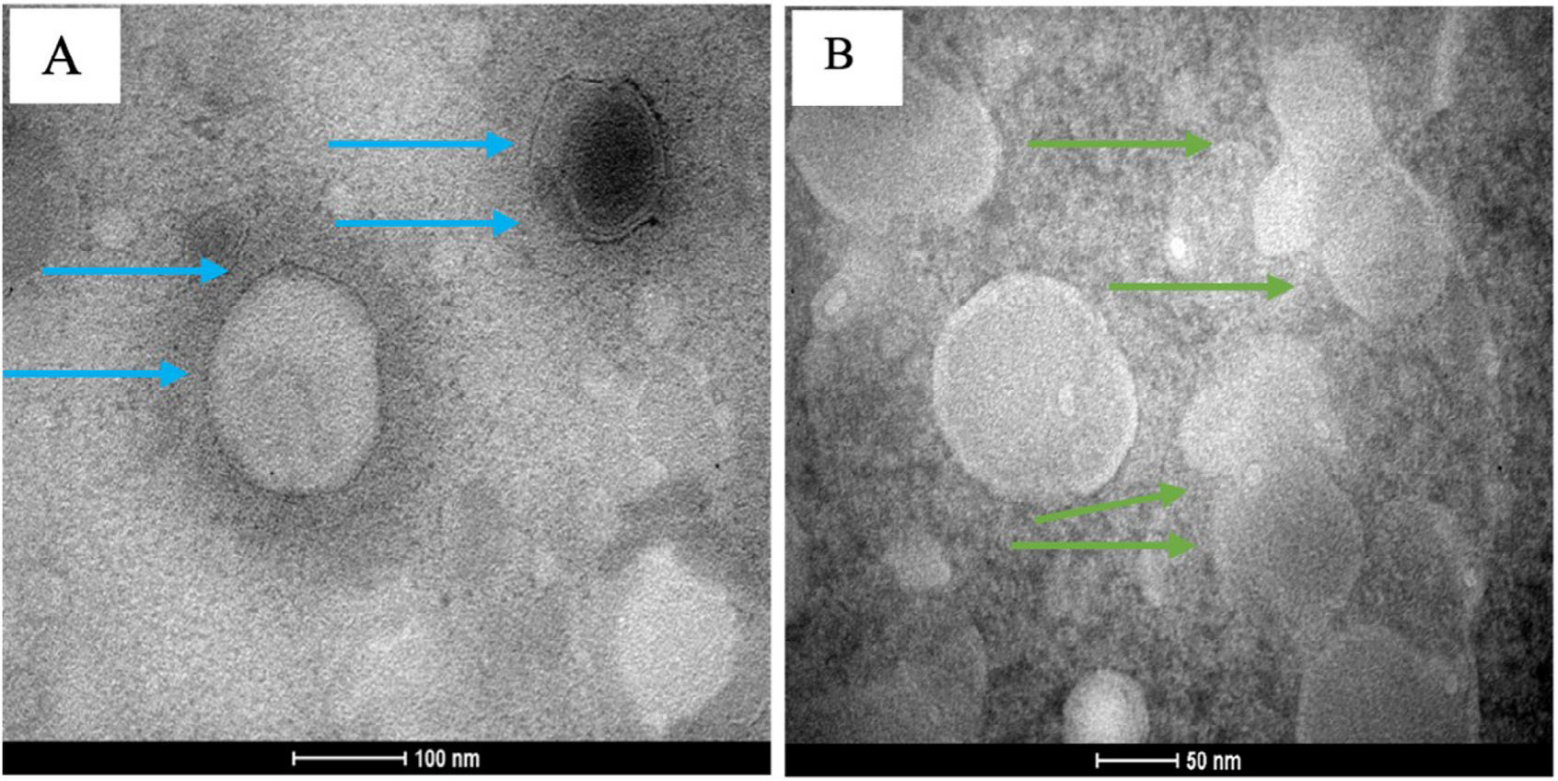
TEM images of the TSL‐LUP01 formulation. (A) Well‐defined circular vesicles characterized by one or more layers (blue arrows) around 100 nm. (B) Homogeneous vesicle distribution with diameters larger than 50 nm, possibly indicating the presence of close bilayers. Green arrows are indications of a possible fusion vesicular process.

Morphological characteristics of the TSL‐LUP01 formulation are available in the transmission electron microscopy (TEM) micrographs (Figure 4). Considering the unfavorable PDI results obtained from the employment Bangham methodology, the morphological study was conducted using samples obtained from the REV method. The obtained images revealed that the adopted liposome preparation procedure results in a system characterized by well‐defined circular vesicles, typically around 100 nm in diameter, as indicated by blue arrows in Figure 4A. Figure 4B is available a set of homogeneous vesicles larger than 50 nm and the possible presence of tight bilayers. The low zeta potential previously observed for this formulation can be confirmed by a possible fusion of two vesicles highlighted by green arrows.

### 2.4 Cellular Uptake Evaluation

The cellular uptake behavior of liposomes by MDA‐MB‐231 cells was studied through the flow cytometry technique using the forward side scatter (FSC) and side scatter (SSC) parameters. The FSC data were used to compare possible changes in cell size. On the other hand, the SSC parameter permits the evaluation of changes in the cell's internal complexity, such as an increase in the number of vesicles or endosomal compartments formed from the liposome uptake and internalization.

**FIGURE 5:**
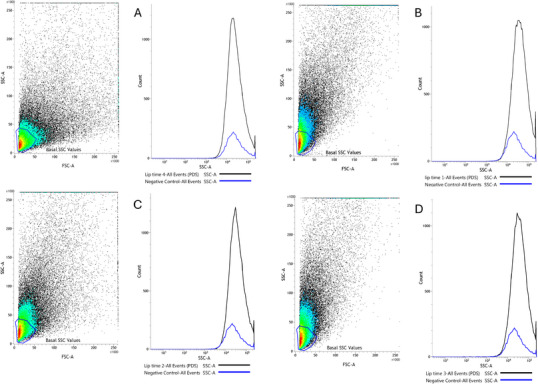
Cellular uptake capacity was studied using the flow cytometry technique. Panels represent MDA‐MB‐231 cells treated with TSL‐LUP01 at 10 µM. Panel (A) describes data from negative control (basal SSC values). Panels (B)–(D) are available data obtained from cells incubated with the TSL‐LUP01 formulation for 20, 40, and 60 min, respectively. The corresponding histograms allow access to the quantitative SSC values. A total of 50 000 events were obtained in each experiment.

The cells were treated with TSL‐LUP01 formulation (lupeol at 10 µM) for 20, 40, and 60 min, and the control group comprised cells treated with sterile saline solution (NaCl 0.9% w/v). The obtained results are available in Figure 5. The data acquired for the negative control are available in Figure 5A and revealed a well‐defined population of healthy cells characterized by low SSC and FSC parameter values. These data were used for gating and defined as basal SSC values. The cells were exposed to the liposomes for 20 min, and afterward, they were washed (four cycles) to remove the physically adsorbed liposomal vesicles. The acquired data are exhibited in Figure 5B. A significant increase in the values of the SSC count parameter can be observed in the density plot graph. Comparatively, the histograms between the control group and treated cells for 20 min revealed a substantial increase in the internal complexity of the treated cells, which is matching with the formation of endosomal vesicles compatible with the liposome internalization processes [31, 32]. The same behavior was observed in cells treated for 40 and 60 min (Figure 5C,D, respectively). It is worth noting that quantitatively, between 20 and 40 min, an observable increase in the SSC values can be seen, which can be attributed to an increase in the number of vesicles formed in the cytosol of the studied cells. After 40 min of incubation, no relevant changes were observed in the SSC versus FSC panel. However, the resulting histogram (area under the curve) shown in Figure 5D showed a slight reduction in the SSC values. This reduction was attributed to the late stage of endosomal pathways, decreasing the number of vesicles.

Considering the different times investigated in this study, the higher quantitative uptake rate was obtained after 40 min of incubation. Previous works available in the literature already demonstrate relevant liposomal uptake by breast cancer cells, including the MDA‐MB‐231 lineage. For example, Okamoto et al. [33] also investigated the uptake behavior of liposomes in MDA‐MB‐231 cells using fluorescent probes. The data obtained revealed that the cells could internalize a significant number of vesicles after 4 h of incubation. On the other hand, Wen et al. [34] observed relevant liposome uptake in the same cell lineage after 60 min.

### 2.5 Cytotoxicity Evaluation

This study evaluated the cytotoxic activity of free lupeol, liposomal formulation (TSL‐LUP01), and controls against MDA‐MB‐231 breast tumor cells. Initially, the cells were treated for 48 h with different concentrations of free lupeol ranging from 5 to 25 µM. The TSL‐LUP01 formulation and controls were tested in concentrations ranging from 1 to 10 µM of lupeol base concentration. From the different data obtained in each test, the IC_50_ was calculated (Prism GraphPad software—version 9.0), and the value found was 44.35 ± 1.03 µM for free lupeol (data not showed). On the other hand, the formulation displayed a significant decline, reaching an IC_50_ of 3.47 ± 0.18 µM, demonstrating a reduction that reached around 12 times. Similar data were found by Zhang that evaluated the free lupeol as an agent for inhibiting the proliferation and migration of MDA‐MB‐231 cells via a novel crosstalk mechanism between autophagy and the epithelial‐mesenchymal transition (EMT) [16]. In the cited study, the authors determined an IC_50_ of 45.67 µM. In fact, comparing the obtained IC_50_ values between the formulation and free lupeol, the encapsulation process has displayed a determinant role for IC_50_ reduction, and this behavior can be associated with differential cell's presentation of lupeol by liposomes, allowing a significant increase in the cytotoxic role of the natural compound here evaluated.

**FIGURE 6:**
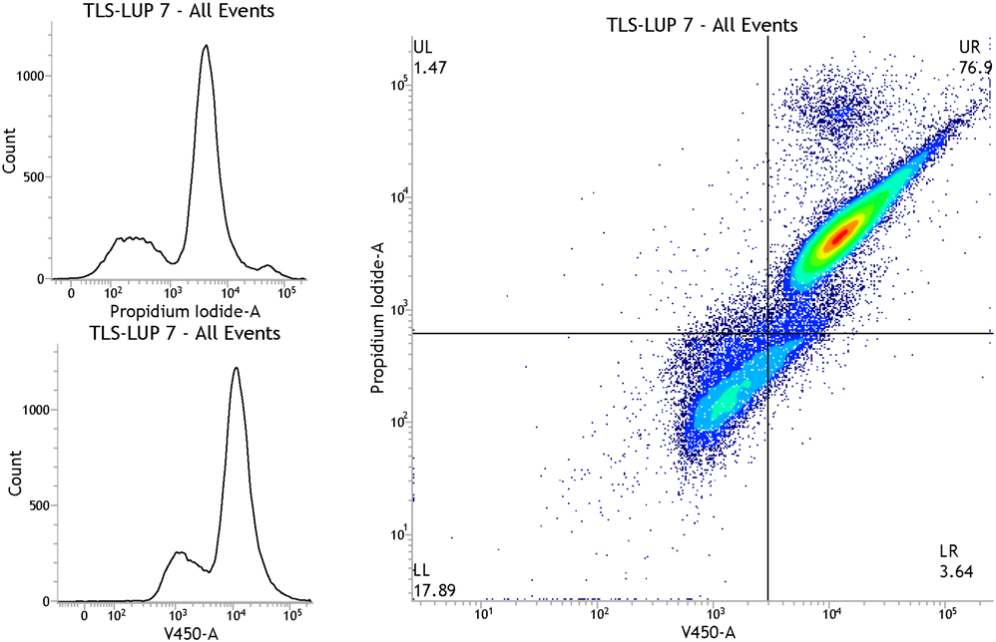
Flow cytometry panels of MDA‐MB‐231 cells treated with TSL‐LUP01 at 5 µM for 48 h. Parts (A) and (B) represent Histplot panels of propidium iodide and Annexin parameters, respectively. (C) Dotplot of propidium iodide (PI) versus Annexin‐V450 (ANNV). LL quadrant = live cells; LR quadrant = early apoptosis; UR = late apoptosis; UL = necrosis. A total of 50 000 events were obtained for each panel.

Cells were treated using different concentrations of TSL‐LUP01 at 5 µM. In the sequence, they were stained with propidium iodide (PI) and Annexin‐V‐450 (ANNV), and the obtained results are available in Figure 6. Flow cytometry panels A and B revealed the histograms obtained for cells treated in terms of PI and ANNV‐450 parameters, respectively. In both graphs, it is possible to identify a well‐defined population of dead cells constituted by high emissions of PI and V‐450. Similarly, concordant data are available in the flow cytometry panel available in Figure 6C, characterized by PI versus ANNV‐450 parameters. When considering the LR, UR, and UL quadrants, the results showed a significant number of dead cells, reaching approximately 82%, characterized by mainly late apoptosis that reached 76.9% of the treated cells. Healthy cells reached only 17.89% (LL quadrant).

The induction of apoptosis is an event that can be achieved in different ways, following two pathways characterized by intrinsic and extrinsic pathways. A significant percentage of cells was determined in late apoptosis, characterized by DNA fragmentation (karyorrhexis), which starts upon completion of nuclear condensation (pyknosis), mediated by both caspase‐dependent and independent routes. Data here found are in accordance with previous studies conducted by Zhang and collaborators and Wang and co‐workers et al., and more recently Mitra et al. for cytotoxicity of lupeol over MDA‐MB‐231 cells [16, 35, 36]. However, our study brings an innovative look demonstrating a singular role of lupeol encapsulation in liposomes, evidenced by a significant increase in the cytotoxicity of lupeol over the studied cells, compared to controls.

Considering the significant population of MDA‐MB‐231 cells that were dead by late apoptosis, we hypothesized the involvement of the AKT/AMPK/mTOR pathway since this can inhibit autophagy and aggravate apoptosis. This hypothesis is based on work described by Meng et al. who identified apoptosis of prostate cancer cells mediated by ursolic acid via the PI3K/Akt/mTOR pathway [10, 37, 38]. However, in future studies, mechanistic studies and specific assays based on Western blot or PCR techniques must be conducted to provide direct evidence regarding this hypothesis and to understand further the role of lupeol cytotoxicity over the breast cancer cells studied.

### 2.6 Cytostatic Capacity Evaluation

**FIGURE 7:**
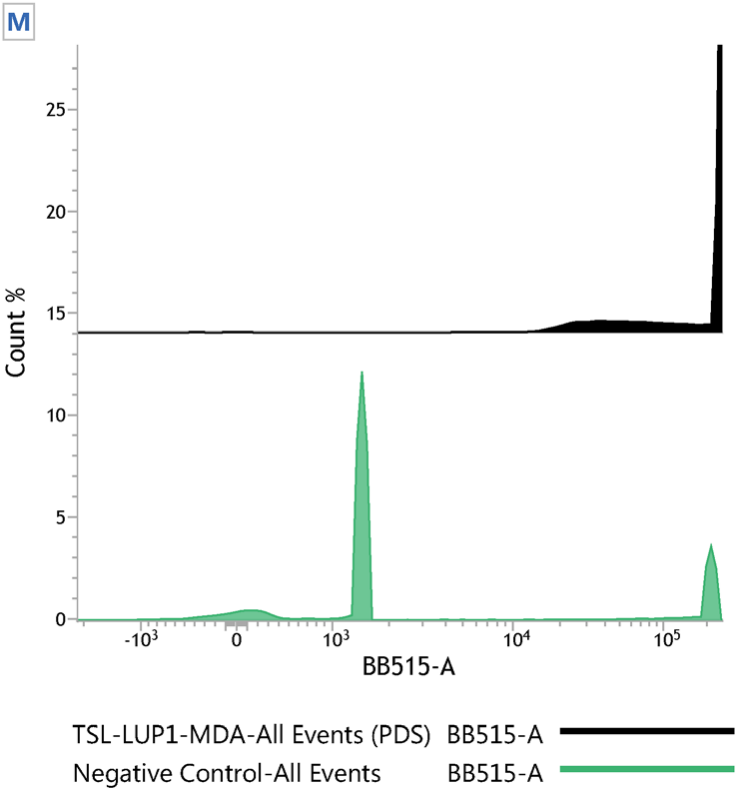
Cytostatic activity evaluation of TSL‐LUP1 and blank liposomes formulations at 10 µM in MDA‐MB‐231 cells treated for 40 min. Control cells were constituted by cells treated with sterile saline solution (NaCl 0.9% w/v).

Carboxyfluorescein diacetate succinimidyl ester (CFSE) has been considered an effective agent for monitoring cellular division processes. As CFSE can covalently bind to long‐lived intracellular molecules, the fluorescent dye can be used to track different generations of cells through cell division in mother and daughter cells [39]. In this sense, the ability of the TSL‐LUP01 system to prevent the proliferation of MDA‐MDB‐231 cells was evaluated by flow cytometry. Cells were exposed to CFSE and treated with TSL‐LUP01 at 10 uM for 2 h. The results are shown in Figure 7. Cells treated with saline showed different peaks due to three different cell generations, as indicated by the letters.

### 2.7 In Vivo Toxicological Profile Evaluation

**FIGURE 8:**
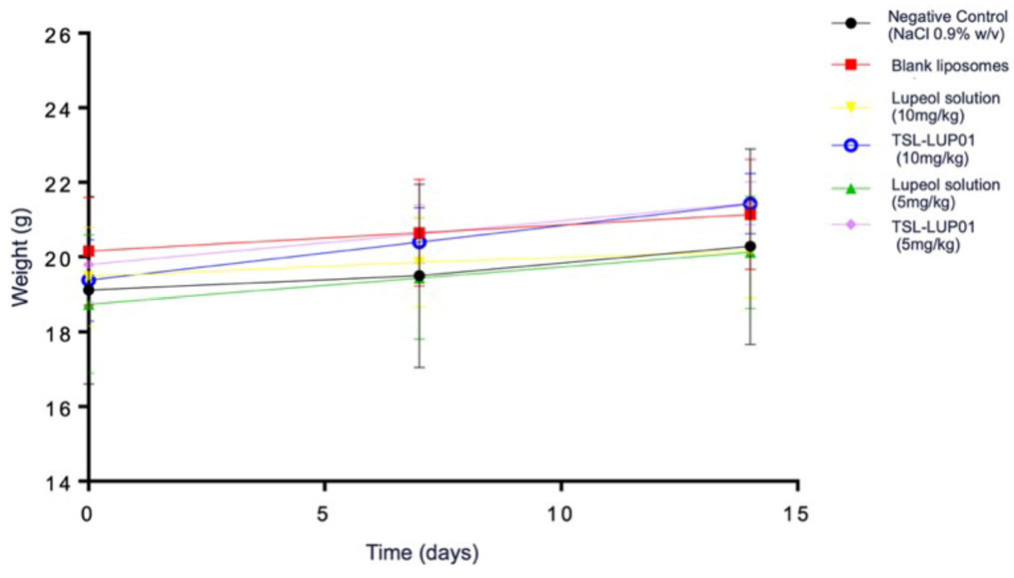
Mice body weight variation study conducted after intravenous administration of different samples constituted by TSL‐LIP01 (10 and 5 mg kg^−1^), lupeol solution (10 and 5 mg kg^−1^), blank liposomes, and NaCl 0.9% (w/v) as a negative control. Statistical differences were evaluated, and nonsignificant differences were found among all samples.

The toxicological profile of the TSL‐LUP01 formulation was studied in healthy female BALB/c mice. The acute toxicity assay was conducted in which a single dose of 5 and 10 mg kg^−1^ TSL‐LUP01 and controls, constituted by blank liposomes (TSL‐BLK), lupeol solution at 780 µg mL^−1^ (vehicle prepared from the use of Tween 20 at 5% (v/v)), saline solution (NaCl 0.9% v/v), and sterile blank vehicle were administrated intravenously to healthy female BALB/c mice (*n* = 6). The data regarding the weight changes during the treatment and hematological and biochemical parameters were evaluated, and the results are available in Figure 8.

The data from the animals treated with lupeol‐liposomes and controls revealed no significant body weight loss (*p* > 0.01). Furthermore, the animals were observed regarding possible patterns in behavioral or clinical changes, morbidity, and mortality (OECD Guideline for Testing of Chemicals, 2001). No significant data were obtained, and all tested animals displayed normal behavior during all the tests. The blank liposomal formulation induced no substantial alterations or negative control as expected. Moreover, it is worth noting that even after the intravenous administration of lupeol solution (5 and 10 mg kg^−1^), no relevant deviation in animals’ behavior or body weight variations was observed, revealing an appropriate and important selective pattern of lupeol for tumor cells. These findings are in accordance with previous data obtained by Saleem and Vijay Avin et al. [17, 40].

Hematological evaluations were also conducted in mice that received the previously described treatments, and the results also revealed no relevant changes among the control and treated groups. The detailed data are available in Table 2. A similar pattern was observed for biochemical investigations (Table 3), where no alterations were found in renal and hepatic parameters. In this work, the systemic toxicity evaluation conducted in mice had the fundamental objective of obtaining initial information regarding the safety of the prepared formulation. As several previously published works have elucidated the nontoxic profile of lupeol through various toxicological evaluations, a concern was raised regarding the different presentation forms of lupeol to the cells. Liposomal vesicles are well‐described systems capable of significantly changing the intracellular pathway of various substances and drugs. Such information is crucial and must be taken before antitumor evaluations, considering the special conditions of mice being debilitated by tumor development.

**TABLE 2:** Hematological parameters of female BALB/c after intravenous injection of saline (CTRL group, blank liposomes, lupeol solution, and TSL‐LUP01 formulation at 5 and 10 mg kg^−1^).

Parameter	CTRL	Blank liposomes	Lupeol sol. 5 mg kg^−1^	Lupeol sol. 10 mg kg^−1^	TSL‐LIP01 5 mg kg^−1^	TSL‐LIP01 10 mg kg^−1^
RBC (cell/mm^3^ x 10^3^)	3.43 ± 0.88	5.36 ± 0.12	5.75 ± 0.37	5.55 ± 0.97	5.85 ± 0.17	4.33 ± 1.62
HGB (g/L)	10.25 ± 2.13	12.4 ± 0.3	13.33 ± 0.75	13.53 ± 0.49	13.38 ± 0.51	13.45 ± 0.64
HCT	23.68 ± 3.94	24.13 ± 0.4	25.78 ± 1.66	26.57 ± 1.08	26.53 ± 0.88	25.3 ± 1.99
WBC (cell/mm^3^ x 10^3^)	3.88 ± 1.04	4.13 ± 0.23	5.23 ± 0.93	7.2 ± 0.98	6.58 ± 1.49	7.75 ± 0.71
Granulocyte (cell/mm^3^ x 10^3^)	0.8 ± 0.39	1.93 ± 0.38	1.8 ± 0.28	2.33 ± 0.67	2.03 ± 0.82	1.48 ± 0.35
Not granulocyte (cell/mm^3^ x 10^3^)	3.08 ± 0.66	2.2 ± 0.61	3.43 ± 1.15	4.87 ± 0.99	4.55 ± 1.02	6.18 ± 0.59
Lymphocytes (cell/mm^3^ x 10^3^)	2.65 ± 0.48	1.43 ± 0.49	2.65 ± 1.37	3.67 ± 0.81	3.45 ± 0.79	4.95 ± 0.51
MYD	0.43 ± 0.26	0.77 ± 0.12	0.78 ± 0.33	1.2 ± 0.2	1.1 ± 0.26	1.23 ± 0.1
PCT	617.75 ± 41.17	634.83 ± 35.36	614.5 ± 56.69	625.00 ± 50.71	649.0 ± 81.76	654.75 ± 44.94

*Note*: Data were expressed as mean (*n *= 6) ± standard deviation of the mean.

Abbreviations: HCT, hematocrit test; HGB, hemoglobin; MYD, myeloid differentiation; PCT, platelets; RBC, red blood cells; RDW, red cell distribution width; WBC, white blood cells.

**TABLE 3:** Biochemical parameters of female BALB/c after intravenous injection of saline (CTRL group; blank liposomes, lupeol solution, and TSL‐LUP01 formulation at 5 and 10 mg kg^−1^).

Parameter	CTRL	Blank liposomes	Lupeol sol. 5 mg kg^−1^	Lupeol sol. 10 mg kg^−1^	TSL‐LIP01 5 mg kg^−1^	TSL‐LIP01 10 mg kg^−1^
AST	97.76 ± 30.04	99.81 ± 17.22	95.46 ± 23.55	103.88 ± 13.77	89.79 ± 11.61	97.94 ± 8.56
ALT	37.39 ± 7.72	31.95 ± 7.92	29.83 ± 5.98	34.78 ± 4.15	28.95 ± 5.10	32.52 ± 4.18
Urea	65.98 ± 16.35	59.4 ± 9.06	61.33 ± 11.65	68.26 ± 11.09	72.05 ± 19.27	70.56 ± 23.16
Creatinine	0.45 ± 0.06	0.50 ± 0.13	0.37 ± 0.06	0.35 ± 0.10	0.33 ± 0.03	0.31 ± 0.05

*Note*: Data were expressed as mean (*n* = 6) ± standard deviation of the mean.

Abbreviations: ALT, alanine aminotransferase; AST, aspartate aminotransferase.

Considering all data obtained in the toxicological evaluation, the tested animals do not reveal relevant toxicological behavior as the data obtained from treated animals are similar to those treated with sterile saline solution. The next stage in animal study must consider biodistribution studies in healthy and tumor‐bearing mice towards comprehension of the internal pathway of the liposomal vesicles produced in this work. These studies, conducted with radiolabeled ^99m^Tc lupeol‐liposomes, for instance, could reveal the nanostructures accumulation profile in tumor tissue and nontarget organs, demonstrating the target‐delivery capacity of the formulation developed, opening possibilities to understand better the delivery strategy adopted.

## 3 Conclusions

Previous studies conducted in the last years have shown the relevant cytotoxicity activity of lupeol against various types of tumor cell lines, including human breast cancer. However, aiming to amplify the understanding of lupeol's role against breast cancer, the present work proposed encapsulating lupeol in a liposomal system to carry the lupeol molecules to the tumor tissues, increasing the delivery process selectively. To achieve this objective, the formulation development process was conducted considering two approaches capable of producing similar formulations with suitable physical, chemical, and morphological properties and important storage stability. After establishing these premises, the next step of the work was to investigate the ability of the liposome‐lupeol system to induce death in human tumor cells with a well‐defined genetic subtype, the TNBC. The results of the in vitro study revealed that cells treated with the liposomal formulation containing lupeol induced apoptosis in many cells, reaching an IC_50_ comparable to those observed by traditional antitumor agents such as cisplatin. These findings follow previously published data, including the same mechanism of death previously elucidated. However, our experiments revealed further data regarding the formulation's cytostatic properties, which could prevent the generation of new descendants of daughter tumor cells. Thus, the authors raised a logical question from these data sets. Is the developed formulation capable of displaying a friendly toxicological profile in healthy animals? Seeking to contribute to answering this question, the systemic toxicological profile of the formulation prepared was evaluated in healthy mice. The different doses revealed normal hematological and biochemical parameters for mice treated with lupeol formulation compared to controls.

Considering the context of the present study, the developed pharmaceutical formulation gathered essential characteristics for tumor treatment and can be considered a potential system against breast cancer.

## 4 Experimental Section

### 4.1 Materials

Dipalmitoylphosphatidylcholine (DPPC), distearoylphosphatidylcholine (DSPC), and distearoyl phosphatidylethanolamine‐polyethylene glycol 2000 (DSPE‐PEG_2000_) were obtained from Lipoid GmbH (Ludwigshafen, Germany). Chloroform was acquired from LabSynth (São Paulo, Brazil). HEPES (4‐(2‐hydroxyethyl)‐1‐piperazine‐ethanesulfonic acid) was supplied by Sigma Chemical Company (São Paulo, Brazil). Fetal bovine serum, streptomycin, and Dulbecco's modified Eagle culture medium (DMEM) were provided by Sigma‐Aldrich (São Paulo, Brazil). Acetonitrile, *tris*‐hydroxymethyl‐aminomethane, and phosphoric acid (85% w/w) were purchased from Merck (São Paulo, Brazil), and ultrapure water, produced from a Smart2Pure 3 UV/UF (Thermo, São Paulo—Brazil), was used in all experiments. BD Horizon BD Horizon Fixable Viable Stain 450 reagent and carboxyfluorescein diacetate succinimidyl ester (CFSE) dye were purchased from BD (São Paulo, Brazil). All chemicals were of ACS or HPLC grade and were used without further purification.

### 4.2 Liposome Preparation

Liposomes were prepared through two different methods: (i) the lipid hydration method, described initially by Bangham and collaborators, and (ii) the REV method [22, 23]. Initially, for both methods, a chloroformic solution of the lipids was prepared. Then 2.25 mL of DPPC (30 mM), 0.6 mL of DSPC (20 mM), and 0.15 mL of DSPE‐PEG_2000_ (10 mM) were transferred to a round bottom flask with a total lipid concentration of 40 mM (molar ratio of 7.5:2.0:0.5, respectively). Lupeol equivalent to 2.34 and 1.17 mM (1 and 0.5 mg mL^−1^‐TSL‐LUP01 and TSL‐LUP05, respectively) was also added to the lipid mixture. The chloroform was removed under reduced pressure, and a thin lipid film was obtained.

For the Bangham preparation methodology, the lipid film was hydrated with 3.0 mL of phosphate buffer saline (PBS), pH 7.4. The obtained dispersion was subjected to vigorous shaking in a vortex, resulting in multilamellar liposomes. For the REV preparation methodology, the lipid film was dissolved in diethyl ether, previously treated with 10 mmol L^−1^ HEPES buffer. After the complete dissolution of the lipids, a solution consisting of PBS, pH 7.4, was added, maintaining a 1:3 ratio of aqueous to organic phase. The resulting mixture was vortexed vigorously at 3000 rpm for 5 min to produce a water‐in‐oil (W/O) emulsion. This emulsion was then subjected to evaporation under reduced pressure to remove the organic solvent and facilitate the formation of lipid vesicles.

In both cases, the resulting multilamellar vesicles were calibrated using a Mini‐Extruder kit (Avanti, Sao Paulo, Brazil) and polycarbonate membranes (06; 0.4; 0.2 µm) in which at least five cycles of extrusion were conducted. Non‐loaded lupeol was separated by dialysis. For this procedure, 10 mL of liposomes was weighed and placed in a cellulose membrane bag (Sigma‐Aldrich, cutoff 20 kDa; São Paulo, Brazil). The system was stirred in 2000 mL of ultrapure water for 48 h at room temperature. The entire contents of the bag were removed and weighed, and PBS buffer (pH 7.4) was added until the same weight was obtained before dialysis. The concentration of lupeol was measured by HPLC.

### 4.3 Physicochemical and Morphological Characterization

The average diameter and the PDI of the vesicles were evaluated by DLS technique using the Nano ZS 90 Zetasizer (Malvern Instruments, Malvern, England) system at 25°C and a fixed angle of 90°. Zeta potential was measured by electrophoretic mobility associated with DLS. All samples were analyzed in triplicate after dilution with HEPES salt buffer pH 7.4 at a 1:30 ratio. Results were expressed as the mean ± standard deviation (SD) of three different batches of each formulation prepared.

A morphological study of the liposomes was carried out using TEM through negative staining. Images were acquired using the Tecnai G2 12 microscope Spirit Biotwin FEI Company system at a voltage of 200 kV (Centro de Microscopia, Universidade Federal de Minas Gerais, Belo Horizonte, Brazil). The liposomes were placed on a formvar‐coated copper grid and stained with a 2% (w/v) phosphotungstic acid solution containing 0.5% (w/v) bovine serum albumin and 0.5% (w/v) saccharose. TEM was performed 24 h after preparing the liposomes to allow for complete drying of the samples.

### 4.4 Encapsulation Efficience Evaluation

The EE study was performed on samples of purified formulations. Triplicates of samples (1 mL) were placed in 15 mL polystyrene tubes containing 5 mL of isopropyl alcohol (HPLC grade) to promote the release of lupeol from the vesicles. The drug concentrations were quantitatively analyzed using the Chromaster Hitachi DAD‐HPLC system.

The mobile phase consisted of a volumetric mixture of acetonitrile, water, and acetic acid (99:1:0.1 v/v)—pH 2.4, in which an isocratic elution was performed. LiChrospher RP‐18 150 × 4.6 mm^2^ column (Merck—São Paulo, Brazil) was used as the stationary phase. The flow rate was 1.0 mL min^−1^, and the injection volume was 20 µL. The temperature of the column oven was set to 25°C. The DAD detection was performed from 210 to 260 nm, and the 215 nm wavelength was monitored. The calibration curve was constructed from six standards at 800, 400, 100, 50, 40, and 10 µg mL^−1^. The standards were prepared from a Lupeol pharmaceutical standard (Certified Reference Material—Merck—São Paulo—Brazil) (*n* = 3). The obtained 3D spectra and chromatograms were processed by Chromaster System Manager software, and the curves were adjusted using linear regression using the least mean square method.

### 4.5 In Vitro Study

The in vitro procedures were conducted on the basis of previous studies published by Vieira et al. [41]. All cell culture media and flow cytometry procedures were conducted in a biosafety level 2 laboratory. Analyses were performed using MDA‐MB‐231 (HTB‐26), a TNBC subtype. DMEM (Sigma Aldrich—São—Paulo Brazil) supplemented with 10% fetal bovine serum, and 2% of an antimycotic antibiotic solution (penicillin, streptomycin, and amphotericin) was used as the culture medium. Cells were incubated (Thermo Fisher Scientific, Asheville, USA) in a controlled atmosphere (5% CO_2_) and humidity at 37°C. After reaching adequate confluence, about 1.0 × 10^8^ cells were transferred to 12‐well cell culture plates and left for 24 h to adhere properly to the plate bottom.

The samples were constituted by sterile free lupeol solution (Tween 20 solution), and TSL‐BLK or TSL‐LUP samples (*n* = 5) were obtained by UV light (253.7 nm—5 W) exposition from a personal sterilizer box for 4 h. The cytotoxicity of samples and controls (positive and negative) consisting of solutions of DMSO (2% v/v) and NaCl (0.9% w/v), respectively, was evaluated through the flow cytometry technique (BD FACsVERSE, San Jose, USA). Free lupeol was tested at 5, 10, 15, and 25 µM. The formulation samples comprised TSL‐LUP01 at 1, 2, 5, and 10 µM. Fixable Viability Stain V450 Reagent KIT was used to differentiate between viable and nonviable cells. The KIT exhibits maximum fluorochrome emission at 450 nm and reacts by covalently binding to amines present on the cell surface and in the intracellular medium. Dead cells show increased fluorescence compared to live cells. For each sample, 50 000 acquisitions were conducted. Early and late apoptosis processes were evaluated through Annexin‐V‐450 dye.

The cytostatic activity of TSL‐LUP01 was evaluated using CFSE (carboxyfluorescein diacetate succinimidyl ester) contrast dye. This dye is effective for monitoring cell division because it can passively diffuse across cell membranes and is cleaved by intracellular esterase in healthy cells. After cleavage, CFSE becomes highly fluorescent and covalently binds to amine groups (proteins). Nonviable cells do not fluoresce. As mitosis is a regular process in viable cells, CFSE is evenly distributed among the daughter cells. As a result, a typical histogram will show different peaks corresponding to different generations of cells, making it easy to track cell generations. Tumor cells were treated for 40 min with TSL‐LUP01 formulation at 10 µM, and controls were constituted by sterile saline solution (NaCl 0.9% w/v). A stock solution of CFSE (10 mM) was transferred to a 15 mL polypropylene centrifuge tube containing 1.0 × 10^7^ cells. The cell suspension was incubated again for 40 min at room temperature. Next, the cells were centrifuged at 1000 rpm, and the resulting pellet was gently mixed and dispersed in a DMEM culture medium. The dispersion was then analyzed using the flow cytometer.

### 4.6 In Vivo Study

The study was based on previous procedures used in the work conducted by Miranda [42]. The study was established using 8‐week‐old female BALB/c mice obtained from CEBIO‐UFMG (Belo Horizonte, Brazil). All animal studies were approved by the Institutional Animal Care and Use Committee (CEUA/UFMG) under the protocol code 204/2023.

A single dose of 5 and 10 mg kg^−1^ TSL‐LUP01 and controls constituted by blank liposomes (TSL‐BLK), lupeol solution at 780 µg mL^−1^ (vehicle prepared from the use of Tween 20 at 5% (v/v); saline solution (NaCl 0.9% v/v), and sterile blank vehicle were administrated intravenously to healthy female BALB/c mice (*n* = 6), weighing around 20 g. Over 14 days posttreatment, the animals were observed for behavioral/clinical changes, body weight, morbidity, and mortality (OECD Guideline for Testing of Chemicals, 2001). After 14 days, the mice were anesthetized with a mixture of ketamine (80 mg kg^−1^) and xylazine (15 mg kg^−1^). The blood was collected via brachial plexus puncture and added into tubes containing anticoagulant (EDTA). Hematological parameters that included hemoglobin, red blood cells (RBC), total white blood cell (WBC) count, neutrophils, lymphocytes, and platelets were evaluated for each group. The parameters were measured automatically using the Hemovet 2300 (São Paulo, Brazil). For biochemical analysis, the blood was centrifuged (3000 rpm, 15 min), and the obtained plasma was frozen at −70°C. Analysis was performed using a Bioplus BIO‐2000 semiautomatic analyzer (São Paulo, Brazil) with commercial kits (Labtest, Lagoa Santa, Brazil). Renal function was evaluated by measuring urea and creatinine; liver function by determining alanine aminotransferase (ALT) and aspartate aminotransferase (AST) activity.

### 4.7 Statistical Analysis

The obtained results were analyzed, when applicable, using ANOVA. The Tukey test measured differences between groups, with *p* values <0.05 considered significant.

## Author Contributions

**Daniel Crístian Ferreira Soares**: funding acquisition, text writing, revision, methodology, experimental, and project coordination. **Daniel Bragança Viana**: experimental methodology, experimental and text writing. **André Luis Branco de Barros**: text writing and revision, methodology, experimental. **Sued Eustáquio Mendes Miranda**: experimental methodology, experimental, and text writing. **Daniela Sachs**: text writing and revision, methodology, experimental. **Eduardo Henrique Martins Nunes**: text writing and revision, methodology, experimental. **Eryvaldo Sócrates Tabosa do Egito**: text writing and revision, methodology, experimental.

## Conflicts of Interest

The authors declare no conflicts of interest.

## Data Availability

The data that support the findings of this study are available from the corresponding author upon reasonable request.

## References

[1] D. Tewari , P. Patni , A. Bishayee , A. N. Sah , and A. Bishayee , “Natural Products Targeting the PI3K‐Akt‐mTOR Signaling Pathway in Cancer: A Novel Therapeutic Strategy,” Seminars in Cancer Biology 80 (2022): 1–17, 10.1016/j.semcancer.2019.12.008. 31866476

[2] M. Á. Curto , E. Butassi , J. C. Ribas , L. A. Svetaz , and J. C. G. Cortés , “Natural Products Targeting the Synthesis of β(1,3)‐D‐Glucan and Chitin of the Fungal Cell Wall. Existing Drugs and Recent Findings,” Phytomedicine 88 (2021): 153556, 10.1016/j.phymed.2021.153556. 33958276

[3] D. H. Dethe , V. Kumar , N. C. Beeralingappa , K. B. Mishra , and A. K. Nirpal , “Synthesis of Polyene Bioactive Natural Products: FR252921 and Vitamin A,” Organic Letters 24 (2022): 2203–2207, 10.1021/acs.orglett.2c00546. 35274951

[4] M. N. Laszczyk , “Pentacyclic Triterpenes of the Lupane, Oleanane and Ursane Group as Tools in Cancer Therapy,” Planta Medica (2009): 1549–1560, 10.1055/s-0029-1186102. 19742422

[5] J. Żwawiak , A. Pawełczyk , D. Olender , and L. Zaprutko , “Structure and Activity of Pentacyclic Triterpenes Codrugs. A Review,” Mini‐Reviews in Medicinal Chemistry (2021): 1509–1526, 10.2174/1389557521666210105110848. 33402080

[6] J. Banerjee , “Bioactive Pentacyclic Triterpenes Trigger Multiple Signalling Pathways for Selective Apoptosis Leading to Anticancer Efficacy: Recent Updates and Future Perspectives,” Current Protein & Peptide Science 24 (2023): 820–842, 10.2174/1389203724666230418123409. 37073661

[7] M. Nistor , D. Rugina , Z. Diaconeasa , C. Socaciu , and M. A. Socaciu , “Pentacyclic Triterpenoid Phytochemicals With Anticancer Activity: Updated Studies on Mechanisms and Targeted Delivery,” International Journal of Molecular Sciences (2023): 12923, 10.3390/ijms241612923. 37629103 PMC10455110

[8] G. Renda , “Immunomodulatory Properties of Triterpenes,” Phytochemistry Reviews 21 (2021): 537–563, 10.1007/s11101-021-09785-x. 34812259 PMC8600492

[9] K. R. Patil , P. Mohapatra , H. M. Patel , et al., “Pentacyclic Triterpenoids Inhibit IKKβ Mediated Activation of NF‐κB Pathway: In Silico and in Vitro Evidences,” PLoS ONE 10 (2015): e0125709, 10.1371/journal.pone.0125709. 25938234 PMC4418667

[10] Y. Meng , Z.‐M. Lin , N. Ge , D.‐L. Zhang , J. Huang , and F. Kong , “Ursolic Acid Induces Apoptosis of Prostate Cancer Cells via the PI3K/Akt/mTOR Pathway,” American Journal of Chinese Medicine (2015): 1471–1486, 10.1142/S0192415X15500834. 26503559

[11] F. Liu , Y. He , Y. Liang , et al., “PI3‐Kinase Inhibition Synergistically Promoted the Anti‐Tumor Effect of Lupeol in Hepatocellular Carcinoma,” Cancer Cell International (2013): 108, 10.1186/1475-2867-13-108. 24176221 PMC3833314

[12] T. Kangsamaksin , S. Chaithongyot , C. Wootthichairangsan , R. Hanchaina , C. Tangshewinsirikul , and J. Svasti , “Lupeol and Stigmasterol Suppress Tumor Angiogenesis and Inhibit Cholangiocarcinoma Growth in Mice via Downregulation of Tumor Necrosis Factor‐α,” PLoS ONE (2017): e0189628, 10.1371/journal.pone.0189628. 29232409 PMC5726636

[13] H. Li , Y. Yu , Y. Liu , et al., “Ursolic Acid Enhances the Antitumor Effects of Sorafenib Associated with Mcl‐1‐Related Apoptosis and SLC7A11‐Dependent Ferroptosis in Human Cancer,” Pharmacological Research (2022): 106306, 10.1016/j.phrs.2022.106306. 35714823

[14] J. Pyo , S. Roh , D. Kim , et al., “Anti‐Cancer Effect of Betulin on a Human Lung Cancer Cell Line: A Pharmacoproteomic Approach Using 2 D SDS PAGE Coupled With Nano‐HPLC Tandem Mass Spectrometry,” Planta Medica (2009): 127–131, 10.1055/s-0028-1088366. 19085751

[15] X.‐D. Li , “Betulin Inhibits Lung Carcinoma Proliferation Through Activation of AMPK Signaling,” Tumor Biology 35 (2014): 11153–11158, 10.1007/s13277-014-2426-7. 25104091

[16] X. Zhang , “Lupeol Inhibits the Proliferation and Migration of MDA‐MB‐231 Breast Cancer Cells via a Novel Crosstalk Mechanism Between Autophagy and the EMT,” Food & Function 13 (2022): 4967–4976, 10.1039/D2FO00483F. 35448900

[17] M. Saleem , “Lupeol, a Novel Anti‐Inflammatory and Anti‐Cancer Dietary Triterpene,” Cancer Letters (2009): 109–115, 10.1016/j.canlet.2009.04.033. PMC2764818 19464787

[18] M. D. Vithana , Z. Singh , and S. K. Johnson , “Regulation of the Levels of Health Promoting Compounds: Lupeol, Mangiferin and Phenolic Acids in the Pulp and Peel of Mango Fruit: A Review,” Journal of the Science of Food and Agriculture (2019): 3740–3751, 10.1002/jsfa.9628. 30723909

[19] J. S. Park , I. U. Rehman , K. Choe , R. Ahmad , H. J. Lee , and M. O. Kim , “A Triterpenoid Lupeol as an Antioxidant and Anti‐Neuroinflammatory Agent: Impacts on Oxidative Stress in Alzheimer's Disease,” Nutrients (2023): 3059, 10.3390/nu15133059. 37447385 PMC10347110

[20] M. Malinowska , B. Miroslaw , E. Sikora , et al., “New Lupeol Esters as Active Substances in the Treatment of Skin Damage,” PLoS ONE (2019): e0214216, 10.1371/journal.pone.0214216. 30921370 PMC6438679

[21] S. Ravichandran and J. Radhakrishnan , “Anticancer Efficacy of Lupeol Incorporated Electrospun Polycaprolactone/Gelatin Nanocomposite Nanofibrous Mats,” Nanotechnology (2022): 295104, 10.1088/1361-6528/ac667b. 35413702

[22] R. Cortesi , “Preparation of Liposomes by Reverse‐Phase Evaporation Using Alternative Organic Solvents,” Journal of Microencapsulation (1999): 251–256, 10.1080/026520499289220. 10080118

[23] A. D. Bangham , M. M. Standish , and J. C. Watkins , “Diffusion of Univalent Ions Across the Lamellae of Swollen Phospholipids,” Journal of Molecular Biology (1965): 238–IN27, 10.1016/S0022-2836(65)80093-6. 5859039

[24] S. C. A. Lopes , M. V. M. Novais , D. S. Ferreira , et al., “Ursolic Acid Incorporation Does Not Prevent the Formation of a Non‐Lamellar Phase in pH‐Sensitive and Long‐Circulating Liposomes,” Langmuir (2014): 15083–15090, 10.1021/la502977j. 25490253

[25] S. Caldeira de Araújo Lopes , M. Vinícius Melo Novais , C. Salviano Teixeira , et al., “Preparation, Physicochemical Characterization, and Cell Viability Evaluation of Long‐Circulating and pH‐Sensitive Liposomes Containing Ursolic Acid,” BioMed Research International (2013): 2013–467147, 10.1155/2013/467147. PMC3747370 23984367

[26] F. Yuan , M. Dellian , D. Fukumura , et al., “Vascular Permeability in a Human Tumor Xenograft: Molecular Size Dependence and Cutoff Size,” Cancer Research 55 (1995): 3752–3756. 7641188

[27] J. Urbanija , N. Tomšič , M. Lokar , et al., “Coalescence of Phospholipid Membranes as a Possible Origin of Anticoagulant Effect of Serum Proteins,” Chemistry and Physics of Lipids (2007): 49–57, 10.1016/j.chemphyslip.2007.06.216. 17662972

[28] G. M. Shashidhar and B. Manohar , “Nanocharacterization of Liposomes for the Encapsulation of Water Soluble Compounds From *Cordyceps sinensis* CS1197 by a Supercritical Gas Anti‐Solvent Technique,” RSC Advances (2018): 34634–34649, 10.1039/C8RA07601D. 35548621 PMC9086942

[29] J. Zhang , “In Vitro and in Vivo Antitumor Effects of Lupeol‐loaded Galactosylated Liposomes,” Drug Delivery (2021), 10.1080/10717544.2021.1905749. PMC8032341 33825591

[30] J. Zhang , H. Liang , H. Yao , et al., “The Preparation, Characterization of Lupeol PEGylated Liposome and Its Functional Evaluation In Vitro as Well as Pharmacokinetics in Rats,” Drug Development and Industrial Pharmacy (2019): 1052–1060, 10.1080/03639045.2019.1569038. 30939950

[31] Y. Ibuki and T. Toyooka , “Nanoparticle Uptake Measured By Flow Cytometry,” Methods in Molecular Biology 926 (2012): 157–166, 10.1007/978-1-62703-002-1_11. 22975963

[32] H. Shin , M. Kwak , T. G. Lee , and J. Y. Lee , “Quantifying the Level of Nanoparticle Uptake in Mammalian Cells Using Flow Cytometry,” Nanoscale (2020): 15743–15751, 10.1039/D0NR01627F. 32677657

[33] Y. Okamoto , K. Taguchi , S. Imoto , V. T. Giam Chuang , K. Yamasaki , and M. Otagiri , “Cell Uptake and Anti‐Tumor Effect of Liposomes Containing Encapsulated Paclitaxel‐bound Albumin Against Breast Cancer Cells in 2D and 3D Cultured Models,” Journal of Drug Delivery Science and Technology (2020): 101381, 10.1016/j.jddst.2019.101381.

[34] X. Wen , J. Li , D. Cai , et al., “Anticancer Efficacy of Targeted Shikonin Liposomes Modified With RGD in Breast Cancer Cells,” Molecules (Basel, Switzerland) (2018): 268, 10.3390/molecules23020268. 29382149 PMC6017468

[35] D. Mitra , D. Saha , and G. Das , “Lupeol Synergizes With 5‐Fluorouracil to Combat c‐MET/EphA2 Mediated Chemoresistance in Triple Negative Breast Cancer,” iScience 26 no. 12 (2023): 108395, 10.1016/j.isci.2023.108395. 38047085 PMC10692664

[36] M. Wang , H. X. Cui , C. Sun , et al., “Effect of Lupeol on Migration and Invasion of Human Breast Cancer MDA‐MB‐231 Cells and Its Mechanism,” Yao Xue Xue Bao = Acta Pharmaceutica Sinica 51 (2016): 558–562. 29859524

[37] J. Xu , Y. Deng , Y. Wang , X. Sun , S. Chen , and G. Fu , “SPAG5‐AS1 Inhibited Autophagy and Aggravated Apoptosis of Podocytes via SPAG5/AKT/mTOR Pathway,” Cell Proliferation 53 (2020): e12738, 10.1111/cpr.12738. 31957155 PMC7046304

[38] R. Wang and W. Hu , “Asprosin Promotes β‐Cell Apoptosis by Inhibiting the Autophagy of β‐cell via AMPK‐mTOR Pathway,” Journal of Cellular Physiology 23 (2021): 215–221, 10.1002/jcp.29835. 32557691

[39] B. J. C. Quah and C. R. Parish , “The Use of Carboxyfluorescein Diacetate Succinimidyl Ester (CFSE) to Monitor Lymphocyte Proliferation,” Journal of Visualized Experiments (2010): 2259, 10.3791/2259. 20972413 PMC3185625

[40] B. R. Vijay Avin , T. Prabhu , C. K. Ramesh , et al., “New Role of Lupeol in Reticence of Angiogenesis, the Cellular Parameter of Neoplastic Progression in Tumorigenesis Models Through Altered Gene Expression,” Biochemical and Biophysical Research Communications (2014): 139–144, 10.1016/j.bbrc.2014.04.090. 24780400

[41] B. B. M. Vieira , I. Lula , N. M. Leão , D. C. F. Soares , J. Fedoce Lopes , and F. B. De Sousa , “Biperiden Hydrochloride/β‐Cyclodextrins Supramolecular System and Its Cytotoxicity Against Lung Adenocarcinoma Cells,” Journal of Molecular Liquids (2023): 122565, 10.1016/j.molliq.2023.122565.

[42] S. E. M. Miranda , “Preclinical Evaluation of L‐Fucoside From Lapachol‐Loaded Nanoemulsion as a Strategy to Breast Cancer Treatment,” Biomedicine & Pharmacotherapy 170 (2024): 116054, 10.1016/j.biopha.2023.116054. 38150876 PMC11878474

